# Establishment of a 3D Co-culture With MDA-MB-231 Breast Cancer Cell Line and Patient-Derived Immune Cells for Application in the Development of Immunotherapies

**DOI:** 10.3389/fonc.2020.01543

**Published:** 2020-08-27

**Authors:** Diana P. Saraiva, Ana T. Matias, Sofia Braga, António Jacinto, M. Guadalupe Cabral

**Affiliations:** ^1^CEDOC, NOVA Medical School, Faculdade de Ciências Médicas da Universidade Nova de Lisboa, Lisbon, Portugal; ^2^Instituto CUF de Oncologia, Lisbon, Portugal

**Keywords:** 3D culture, spheroids, breast cancer, tumor immune microenvironment, drug screening, immunotherapy

## Abstract

3D cell culture including different cell types, such as immune cells, is a representative platform that mimics the tumor microenvironment. Here we disclose an easy-to-handle 3D co-culture protocol using a scaffold-free technique with the breast cancer cell line MDA-MB-231 and breast cancer patient-derived immune cells from peripheral blood. The method presented is simple, less time-consuming and less expensive when compared to other 3D techniques. Additionally, this is an optimized protocol for the establishment of a 3D system for this cell line, which is normally seen as challenging to spontaneously form spheroids. The addition of patient-derived immune cells to the cancer cells' spheroid allows the study of the crosstalk between both cell types, as well as the assessment of individual therapeutic approaches to intensify the antitumor immune response. In fact, with this model, we observed that patients' immune cells exhibit a wide range of antitumor responses and we further demonstrated that it is possible to manipulate the less effective ones with a canonical stimulus, as a proof-of-concept, in order to improve their ability to lower the viability of tumor cells. Therefore, this platform could be applied for a personalized immune-based drug screening, with results after a maximum of 10 days of culture, in order to develop more tailored breast cancer treatments and ameliorate patients' survival rate.

## Introduction

Breast cancer remains the main leading cause of cancer-related deaths in women worldwide (1). A growing body of evidence supports that breast cancer prognosis may be related to the individual immune system functional status (2), which may explain, at least in part, why some patients respond to the established treatments, whilst others do not (3). Indeed, tumors have mechanisms to escape the immune system surveillance, which includes the release of anti-inflammatory cytokines (such as TGF-β or IL-10), the activation of immune checkpoints and the recruitment of suppressive immune cell types (4).

To overcome this immune escape, immunotherapies can be implemented. In fact, increasing numbers of immunotherapies for cancer treatment have been coming to the market over the past decade, revolutionizing the way many cancers are treated, especially lung cancer and melanoma, which drastically improved patients' outcome (5, 6). Nevertheless, patient response rates can fluctuate among cancers and within cohorts with the same malignancy, for reasons that are not so well understood, but that should include different immune competency, antigen specificity and expression levels (7, 8). For other common cancers, including breast cancer, immunotherapy, in particular with the most known FDA approved immune checkpoint blockers, such as PD-1 (programmed cell death protein 1), PD-L1 (programmed death-ligand 1), and CTLA-4 (cytotoxic T-lymphocyte-associated protein 4), have been shown a low success rate (9, 10), with only Atezolizumab (anti-PD-L1) being approved for the treatment of metastatic triple negative breast cancer (TNBC).

Bearing in mind that specific reactivation of patients' immune system is one of the major goals to improve breast cancer treatment, though the mechanisms refraining the antitumor immune responses might be different from patient to patient, it is important to further develop immunotherapies that work in individual patients. The tumor immune microenvironment (TIME) has been widely studied in breast cancer, in order to find new personalized therapeutic strategies based on immune-modulators that could aid the standard chemotherapeutic options and improve breast cancer patients' survival (11). In order to screen novel therapies, the development of new platforms that reliably mimic tumor structures should be addressed. Mice models hardly mimic the treatment resistance and the immune response observed in patients, and human models consisting of monolayer cultures (2D) poorly recapitulate the complexity of the tumor environment. Thus, many studies have been emphasizing the use of human 3D cultures to study with more accuracy the efficacy of different anticancer treatments. Additionally, their formation and assessment are less time-consuming when compared to animal models (12). 3D *in vitro* cell culture offer more efficient cell-cell and cell-matrix interactions, which influences cell structure, cell signaling, cell adhesion and mechano-sensing. Moreover, the diffusion of oxygen and nutrients is hampered to the hypoxic core of the structure and produced metabolites are also poorly diffused from the core to the surface of the 3D structure (13, 14). Finally, 3D systems allow for the addition of different cell types, creating a multicellular structure, necessary for the study of the TIME and alternative therapeutic options (15, 16).

Nevertheless, there are multiple ways to develop a 3D culture system. Here, we disclose a protocol for a liquid overlay technique, which consists in coating the plate with a non-adhesive component to enhance cell-cell interaction, allowing a spontaneous 3D spheroid formation (17). This approach is scaffold-independent, easy to work and handle, reproducible and less expensive, when compared to the use of commercial low adherence plates or even scaffold-based approaches that use Matrigel or collagen, for instance (17, 18). Another advantage is the low number of cells needed to form the 3D structure, which is a beneficial characteristic when working with patient-derived cells. Although other protocols have been established for the formation of breast cancer spheroids with immune cell invasion, here we reveal a protocol specifically for MDA-MB-231 cell line, which is often seen as a difficult cell line to spontaneous form spheroids without scaffolds (19, 20). The employment of this cell line in drug-screening platforms is particularly relevant because it is a highly aggressive, invasive and poorly differentiated TNBC cell line. TNBC lacks the estrogen and progesterone receptor (ER and PR) expression, as well as the human epidermal growth factor receptor 2 (HER2), currently having limited treatment options (21). Interestingly, this cell line, differently from other common breast cancer cell lines, such as MCF-7, express high levels of the immune checkpoint PD-L1 (22), which can bind to its receptor PD-1 at the surface of effector T lymphocytes, therefore acting as a brake and impairing the activation and the assemble of a proper antitumor response. PD-L1, a key component of the tumor immune evasion mechanisms, is indeed highly expressed in many breast cancer patients' tumors, especially in cases with poorly activated tumor-infiltrating effector T lymphocytes (3).

Additionally, with this protocol, it is possible to perform a co-culture in a 1:1 ratio of tumor cells to immune cells and observe the effect of the later in the tumor. This is an advantage when comparing to other protocols with a 10:1 ratio of immune cells to tumor cells (23), since it is more representative of *in vivo* immune cells infiltration into the tumor microenvironment, as we observed previously (3).

Thus, besides describing in detail the protocol employed for the establishment of this 3D system, we also demonstrate the utility of this allogeneic system−3D co-culture of MDA-MB-231 cell line with breast cancer patient-derived immune cells—in the development of novel therapies, as treating the immune cells with an external canonical stimulus could improve their cytotoxic activity against the tumor cells. We believe that this system can become extremely useful to test, in a simple and economic fashion, several clinical grade immune-modulators that alone or in combination with chemotherapeutic compounds could improve the anti-tumor response of individual breast cancer patients.

Likewise, this protocol also has the advantage of possible modifications to include different cell types such as fibroblasts or even tumor cells, matching the immune cells, to build an autologous system.

## Materials and Equipment

### Materials/Reagents

1. MDA-MB-231 (ATCC^®^ HTB-26™)
2. Dulbecco's Modified Eagle Medium (DMEM, Biowest, catalog number: L0102-500), store at 4°C
3. Fetal Bovine Serum (FBS, Sigma Aldrich, catalog number: F9665-0500), store at−20°C
4. Penicillin/Streptomycin (Pen/Strep, GE Healthcare, catalog number: SV30010), store at −20°C
5. TrypLE (Gibco, catalog number: 12605028), store at 4°C
6. RPMI-1640 (Gibco, catalog number: 21875-034), store at 4°C
7. Trypan blue (GE Healthcare, catalog number: SV30084.01), store at room temperature
8. 6-well plates for tissue culture (VWR, catalog number: 734-2323)
9. Ficol (Biocol, Merck Millipore, catalog number: L6715), store at room temperature
10. PBS 1X (see recipes), store at room temperature
11. Dimethyl sulfoxide (DMSO, Sigma, catalog number: D5879), store at room temperature
12. Blood collection tubes with EDTA (BD Biosciences, Vacutainer EDTAK2, catalog number: BD367525)
13. Pasteur pipettes (Sarstedt, catalog number: 86.1171.001)
14. Agarose (Invitrogen, catalog number: 16500–500), store at room temperature
15. Distilled water (dH_2_O)
16. 96-well plates with round bottom (Sigma Aldrich, catalog number: M9436-100EA)
17. 15 mL falcons (VWR, catalog number: 525-0604)
18. 50 mL falcons (VWR, catalog number: 525-0610)
19. 1.5 mL microtubes (Enzifarma, catalog number: P10202)
20. Optional: Cell Trace CFSE proliferation kit (Invitrogen, catalog number: C34554), store at −20°C
21. Optional: CellTracker™ Orange CMTMR (5- (and-6)–(((4-chloromethyl)benzoyl)amino)tetramethylrhodamine) (Invitrogen, catalog number: C2927), store at −20°C
22. Optional: Phorbol 12-myristate 13-acetate (PMA, Sigma Aldrich, catalog number: P1585), store at −20°C
23. Optional: ionomycin (Merck, catalog number: 407952), store at −20°C
24. Optional: BD Horizon^TM^ Fixable Viability Stain 450 (BD Biosciences, catalog number: 562247), store at −20°C
25. Optional: FlowFix (Polysciences, catalog number: 25085-1), store at 4°C
26. Optional: mouse anti-human CD45-PercP (Biolegend, catalog number: 304026), store at 4°C
27. Optional: Human IFN-gamma ELISA MAX™ Standard (Biolegend, catalog number: 430101), store at 4°C.

### Equipment

1. Micropipettes
2. Automatic pipettes
3. Multichannel pipettes
4. Centrifuge
5. Biosafety cabinet with vertical flow (ESCO, LA2-4A1)
6. 37°C CO_2_ incubator
7. Water bath able to reach 90°C
8. Hemocytometer
9. Autoclave
10. Fluorescence microscope (Zeiss, model Axiovert 40 CFL) or confocal microscope (Zeiss, model LSM710)
11. Flow cytometer (BD, FACS Canto II).

### Software

1. GraphPad Prism v.7
2. FlowJo v.10
3. Image J 1.52c.

### Recipes

1. PBS 1X
  - Prepare 2 L PBS stock (10X) by adding:
    160 g NaCl
    4 g KCl
    28.8 g Na_2_HPO_4_
    4.8 g KH_2_PO_4_
  - Dissolve in 1800 mL MilliQ H_2_O and mix well.
  - Adjust the pH to 7.4.
  - Add MilliQ H_2_O until a total volume of 2 L.
  - Prepare PBS 1X by adding:
    100 ml PBS 10X
    900 ml MilliQ H_2_O
  - Mix well and send it to autoclave.
2. Supplemented DMEM
  - Add 10% of FBS and 1% of Pen/Strep to total DMEM.
3. Supplemented RPMI
  - Add 10% of FBS and 1% of Pen/Strep to total RPMI.

### Methods

#### Establishment of 3D Co-cultures

##### 2D culture of MDA-MB-231 cell line

1. Thaw MDA-MB-231 cryovial (with 1 mL of cells suspended in FBS with 10% DMSO) by placing rapidly in a water bath at 37°C.
  *Note: remove the cryovial from the water bath immediately when only a small fragment of ice is observed*.
2. With a Pasteur pipette add the cell suspension to a falcon with 4 mL of supplemented DMEM, drop by drop.
  *Note: the supplemented cell medium must be at 37*°*C before use*.
3. Centrifuge at 200 g for 5 min.
4. Count the cells in a hemocytometer with trypan blue to exclude dead cells and resuspend the desired concentration in supplemented DMEM.
5. Plate the cells in a 6-well plate (2 mL of medium per well) and incubate in a humidified environment at 37°C, 5% CO_2_.
6. When cells reach an 80–90% confluency, pass the cells by removing the medium, wash with PBS 1X and add 500 μL of TrypLE to each well and incubate at 37°C.
7. When cells begin to detach, add the same volume of supplemented DMEM and centrifuge at 200 g for 5 min.
8. Remove the supernatant, add supplemented DMEM and seed in a new 6-well plate.

##### Isolation of peripheral blood mononuclear cells (PBMCs)

1. Dilute whole blood in PBS 1X (1:1 ratio) in a 50 mL falcon and mix well.
2. In a new 50 mL falcon with Ficol, layer the blood solution, with a Pasteur pipette, on top of Ficol in a 3:5 ratio (Ficol:blood).
  *Notes: perform this step very gently to avoid disturbing the Ficol layer. The Pasteur pipette can be placed in a 45*° *angle and the blood solution can be released directly on the wall of the falcon*.
3. Centrifuge at 1,000 g for 30 min without brake.
4. With a Pasteur pipette carefully remove the PBMCs layer (between the plasma and the Ficol) to a new 50 mL falcon.
5. Add 20 mL of PBS 1X and centrifuge at 300 g for 5 min.
6. Remove the supernatant, add 10 mL of PBS 1X and centrifuge at 200 g for 5 min.
7. Count the number of PBMCs in a hemocytometer with trypan blue to exclude the dead cells.
8. Freeze the cells in FBS with 10% DMSO in a concentration of ~5 × 10^6^ cells/mL per cryovial.
  *Note: when adding the freezing medium (FBS with DMSO) do it very gently to avoid loss of cell viability in the presence of DMSO*.
  Store at −80°C until further use.

##### 3D Co-culture Assembly

**Figure 1:**
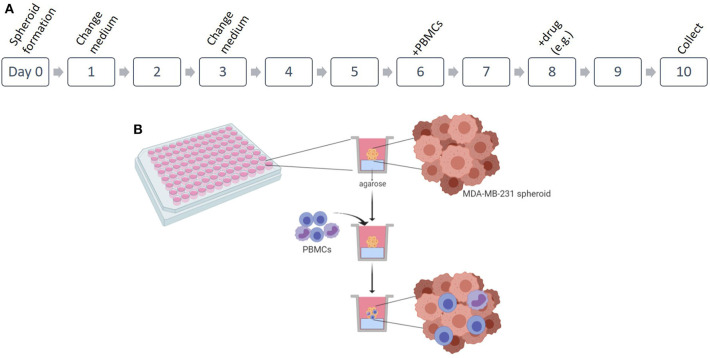
Establishment of 3D co-culture of breast cancer cell line and patient-derived immune cells. **(A)** Scheme of the protocol for the 3D *in vitro* spheroids of MDA-MB-231 cell line in co-culture with patient-derived peripheral blood mononuclear cells (PBMCs). **(B)** Representation of the 3D culture in 96 well plates and the infiltration of the PBMCs. Figure performed in Biorender.

The 3D co-culture of MDA-MB-231 with patient-derived PBMCs follows a series of consecutive steps, which can be observed in the scheme in Figure 1. *Note: check spheroid development daily in an optical microscope to assess if spheroid is formed or to analyze possible contaminations*.

**Figure 2:**
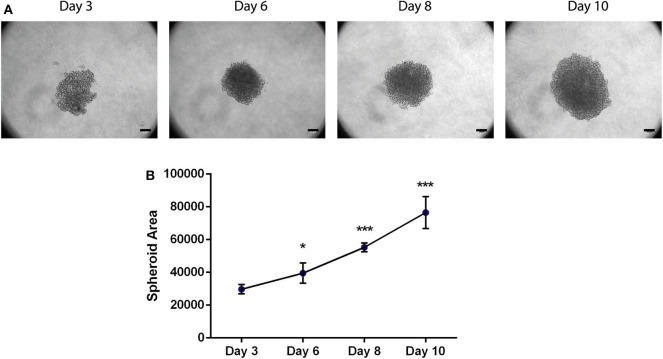
Area of the 3D spheroids with MDA-MB-231 breast cancer cell line increases with time. **(A)** Bright-field images of MDA-MB-231 spheroids at days 3, 6, 8, and 10. Scale bars−50 μm. **(B)** Spheroid area of MDA-MB-231 spheroids at day 3 (*n* = 4), day 6 (*n* = 7), day 8 (*n* = 4), and day 10 (*n* = 10). Data is represented as mean ± SD, *p < 0.05, ***p < 0.001 (paired *t*-test).

**Figure 3:**
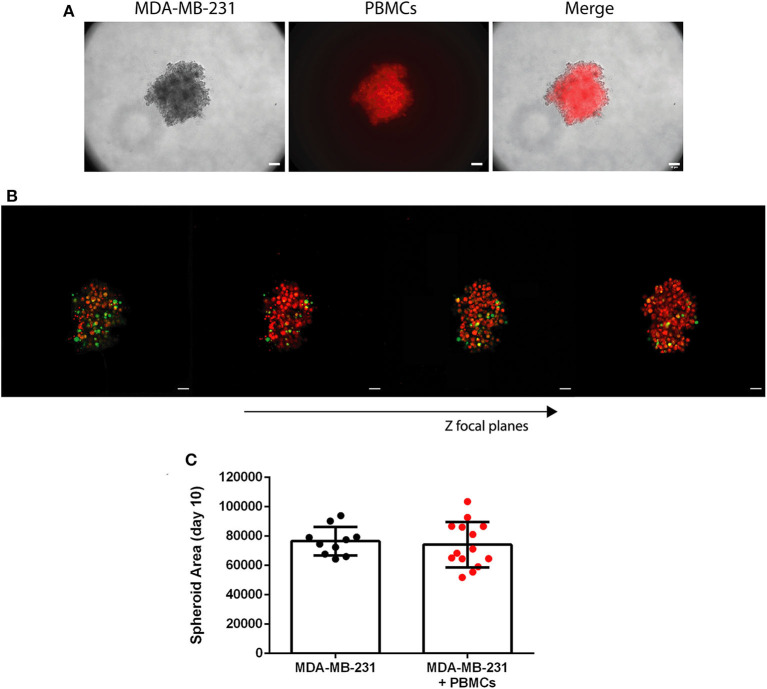
Patient-derived immune cells are able to infiltrate the 3D spheroid of MDA-MB-231 breast cancer cell line. **(A)** Bright field of MDA-MB-231 spheroid (left panel), patient-derived PBMCs stained in red with a cell tracer dye (middle panel) and the two photos merged (right panel), at day 7 of the culture (24 h after the addition of the PBMCs); 10x objective, scale bar 50 μm. **(B)** Confocal images of 3D spheroids of MDA-MB-231 cell line (stained in green with CFSE) and patient-derived PBMCs (stained in red with a cell tracer dye). Stills were acquired with a 10x objective for different Z focal planes, to demonstrate the immune infiltration in a 3D structure. Images were assembled with Image J; scale bar 50 μm. **(C)** Spheroid area of MDA-MB-231 cells in monoculture (*n* = 10) and in co-culture with breast cancer patient-derived PBMCs (+PBMCs, *n* = 14) at day 10 of the culture. Data are represented as mean ± SD.

1. Day 0:
  - Prepare in advance 1.5% agarose in dH_2_O and autoclave the solution.
    *Note: a new agarose solution should be prepared every time a new 3D culture protocol is initiated*.
  - After the autoclave and the solidification of the agarose, place the solution in a water bath at 90°C for 1 h.
    *Note: alternatively, use the agarose directly from the autoclave, letting the solution cool down without solidifying*.
  - Add 50 μL of agarose per well with a multichannel pipette in a 96-well plate with round bottom.
    *Notes: perform this step rapidly as agarose can solidify. If the agarose starts to solidify when pipetting, place it again in the water bath. Avoid the formation of bubbles that will compromise spheroid formation*.
  - Allow to cool down for 20 min.
  - Add 1,000 MDA-MB-231 cells in 200 μL of supplemented DMEM per well.
  - Incubate in a humidified environment at 37°C, 5% CO_2_.
2. Day 1:
  - Remove 100 μL of medium per well and discard.
    *Note: remove the medium by placing the pipette tip with a 45*° *angle and gently aspirate from the top of the well, without touching the spheroid(s)*.
  - Add 100 μL of fresh supplemented medium (see recipes) per well.
  - Incubate in a humidified environment at 37°C, 5% CO_2_.
3. Day 3:
  - Remove 100 μL of medium per well and discard (see note in 2.a).
  - Add 100 μL of fresh supplemented medium (see recipes) per well.
  - Incubate in a humidified environment at 37°C, 5% CO_2_.
4. Day 6:
  - On this day the spheroid must be fully formed (Figure 2) and co-culture can be assembled.
  - Thaw PBMCs by placing the cryovial rapidly in a water bath at 37°C (see note in 2D culture section).
  - With a Pasteur pipette add the cell suspension to a falcon with 4 mL of supplemented RPMI, drop by drop.
  - Centrifuge at 200 g for 5 min.
  - Count the cells in a hemocytometer with trypan blue to exclude dead cells and resuspend the cells with the desired concentration in supplemented RPMI.
  - Remove 100 μL of medium per well in the 96-well plate and discard (see note in 2.a).
  - Add 100 μL per well of supplemented RPMI containing 1000 PBMCs to perform a 1:1 ratio co-culture.
  - Incubate in a humidified environment at 37°C, 5% CO_2_.
    *Note: if using PBMCs stimulated with PMA and ionomycin, add the same percentage of DMSO (used to prepare ionomycin) to the wells without PBMCs or with non-stimulated PBMCs*.
5. Day 7:
  - At this stage, PBMCs are already infiltrated in the 3D spheroid structure (Figure 3).
6. Day 8:
  - Remove 100 μL of medium per well and discard (see note in 2.a).
  - Add 100 μL of fresh supplemented RPMI (see recipes) per well (at this stage, chemical and/or biological compounds can be added—see drug screening section).
    *Note: if the compounds are diluted in dimethyl sulfoxide (DMSO), keep the same DMSO concentration in all the wells*.
  - Incubate in a humidified environment at 37°C, 5% CO_2_.
7. Day 10:
  - At this time point, the area, viability and other parameters of the spheroids and the molecules present in their supernatant can be analyzed.

#### Techniques to Characterize the Spheroids

This system allows the determination of several parameters of the whole spheroids. First, we used Imaging techniques, to determine the spheroid area and, by employing two cell tracers (one red and one green), identify both cell types (tumor and patient-derived PBMCs) and address the infiltration of the PBMCs in the 3D tumor-like structure. Although not employed here, immunofluorescence can also be performed if desired, to detect distinct cellular markers, such as those involved in tumor cell apoptosis, proliferation, or PBMCs' activation, using confocal microscopy. Here, we have opted for Flow Cytometry and ELISA to address the effect of the PBMCs, with or without previous stimulation, in the breast cancer cell line viability. Flow Cytometry was used to determine the viability of both cell types; however, this technique is also useful to pinpoint surface and/or intracellular markers to distinguish cell populations and to assess levels of immuno-activation or -suppression, for instance. ELISA assays of the co-culture supernatant were performed to quantify secreted IFN-γ, which is one of the main cytotoxicity-related molecules released by effector T cells. This technique can also be performed to analyze the level of other soluble molecules, including cytokines, depending on the objective of the experiment. Here we focused particularly on these methods; nevertheless, we envisage several others that could be employed to analyze these 3D co-cultures, such as western blot, to assess different markers in the whole spheroid (independently of the cell population), real-time qPCR, to address gene expression, and fluorescence activated cell sorting (FACS) to separate different cell populations in order to further study them individually.

##### Flow Cytometry

Different cell surface or intracellular markers can be assessed by Flow Cytometry after dissociation of the spheroid and incubation with target fluorescent monoclonal antibodies. Additionally, the viability of the spheroid cells could be determined using fluorescent viability dyes. Here we used a viability dye to assess the effect of the addition of unstimulated/stimulated breast cancer patient-derived PBMCs in the tumor cells. Additionally, to distinguish tumor and immune cells, the pan-leukocyte marker CD45 was used.

**Figure 4:**
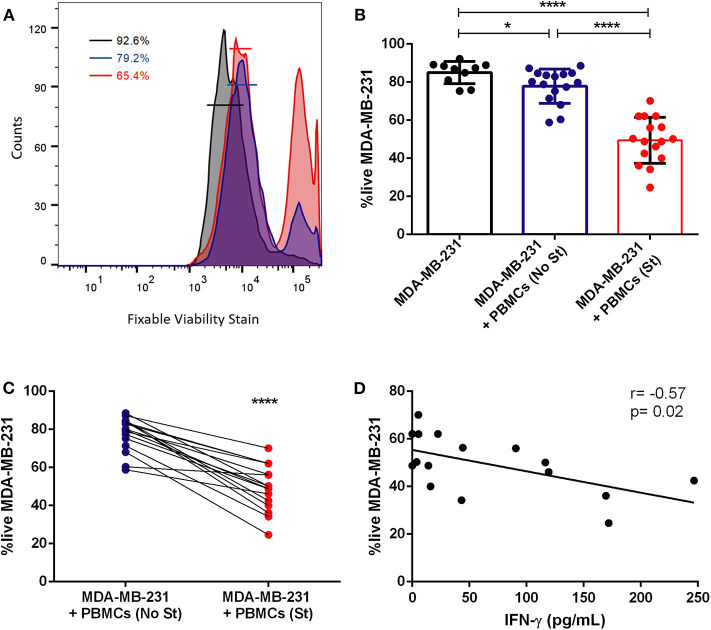
Previous stimulation increases the cytotoxicity of patient-derived immune cells against tumor cells. **(A)** Representative histogram of the flow cytometry analysis of the viability of MDA-MB-231 in monoculture (black line), in co-culture with non-stimulated PBMCs (blue line) and in co-culture with previously stimulated PBMCs (red line); the percentage of live cells in each condition is represented. **(B)** Percentage of viable breast cancer cells (MDA-MB-231) in monoculture (black bar, *n* = 10), in co-culture with PBMCs (+PBMCs (No St), blue bar, *n* = 16) and in co-culture with PMA/ionomycin stimulated PBMCs (+PBMCs (St), red bar, *n* = 16). **(C)** Data on the viable tumor cells in co-culture (same as in B) represented as connected dots, to highlight the effect of PBMCs stimulation in individual cases. **(D)** Correlation (Spearman r) between the viability of the MDA-MB-231 cell line and the production of IFN-γ, detected in the co-culture supernatant and quantified by ELISA. Data is represented as mean ± SD. Mann-Whitney test was applied in **(B)** and a paired *t*-test was used in **(C)**, *p < 0.05, ****p < 0.0001. All the wells have the same quantity of DMSO, as this reagent was used to prepare ionomycin.

1. Collect the spheroids at day 10 by pipetting the total 200 μL from each well.
  *Note: perform this step with a P1000 micropipette*.
2. Transfer the spheroid to a 1.5 mL microtube and disrupt the structure by pipetting up and down several times.
  *Note: use at least 6 spheroids per condition*.
3. Centrifuge at 115 g for 5 min.
4. Remove the supernatant and resuspend the pellet in PBS 1X.
5. Add 1 μL of BD Horizon^TM^ Fixable Viability Stain 450 per mL of PBS 1X.
  *Note: leave at least 100* μ*L of cell suspension for the unstained condition*.
6. Incubate in the dark, at 4°C for 20 min.
7. Centrifuge at 300 g for 5 min.
8. Remove the supernatant and resuspend in PBS 1X
9. Add 2 μL of anti-CD45-PercP per 100 μL of PBS 1X.
  *Note: leave at least 100* μ*L of cell suspension in viability stain for fluorescence minus one control*.
10. Incubate 15 min at room temperature in the dark.
11. Centrifuge at 300 g for 5 min.
12. Remove the supernatant and add PBS 1X or FlowFix.
13. Analyze in the flow cytometer.
14. Perform data analysis in FlowJo (see Figure 4A).

##### ELISA

Cytokines or other molecules released to the extracellular milieu can be quantified by ELISA (see Figure 4D).

1. Collect the spheroids at day 10 by pipetting the total 200 μL from each well (see note in flow cytometry section).
2. Transfer the spheroid to a 1.5 mL microtube. *Note: use at least 6 spheroids per condition*.
3. Centrifuge at 115 g for 5 min.
4. Remove the supernatant to a new 1.5 mL microtube.
5. Centrifuge at 375 g for 10 min to remove any cellular debris.
6. Collect the supernatant to a new 1.5 mL microtube.
7. Aliquot and store at −80°C or use immediately.
8. Perform the ELISA assay following the manufacturer's instructions.

##### Imaging

Image acquisition can be performed in bright field or fluorescence. Here, we have used bright field snapshots to determine the spheroid area in Image J software. However, other spheroid parameters could also be determined, such as the diameter and volume based on the same images. To diminish the operator bias, an automatic threshold was applied to the image (Image—Adjust—Threshold) and quantification of the area was performed in the appropriate tear-down menu (Analyze—Measure).

For fluorescence, one of the two distinct staining methods—cell tracers or immunostaining, could be employed and although here we just used cell tracers, we opted to described both:

1. Cell tracers (see Figure 3).
  - Following the manufacturer's instructions, resuspend MDA-MB-231 cells in PBS 1X before the start of the 3D culture.
  - Stain with 5 mM of Cell Trace CFSE proliferation kit for 20 min in the water bath at 37°C.
  - Add supplemented DMEM and incubate in the water bath at 37°C for 5 min.
  - Centrifuge at 115 g for 5 min.
  - Resuspend in supplemented DMEM in the desired concentration.
  - Perform the 3D culture as above.
  - Place the PBMCs in unsupplemented RPMI and add 5 mM of CellTracker™ Orange CMTMR.
  - Incubate in the water bath at 37°C for 30 min.
  - Centrifuge at 115 g for 5 min.
  - Remove the supernatant and add fresh supplemented RPMI.
  - Perform the co-culture as explained above.
  Acquire images of the spheroids in the co-culture directly in the plate wells, after 24 h, in a fluorescence or confocal microscope with lasers 488 and 568.
  Here, stills were acquired with a 10x objective for different Z focal planes, to demonstrate the immune infiltration (red cells) in the 3D tumor-like structure (green). Images were assembled in Image J.
2. Fixing followed by immunostaining (not employed here)
  - At the end of the co-culture, remove the medium.
    *Note: be very careful to avoid disturbing the spheroid*.
  - Add 200 μL of 4% paraformaldehyde (PFA) and incubate for 20 min.
    *Note: alternatively, remove 100* μ*L of medium and add 100* μ*L of PFA. Wait 5 min to stabilize the spheroid, remove another 100* μ*L of medium with PFA and add 100* μ*L of fresh PFA and incubate for 20 min*.
  - Remove the PFA and perform the staining.
    *Note: it is recommended to perform the staining on the same plate and observe in the microscope with the same plate to minimize spheroid destabilization*.

#### Statistical Analysis

Statistical analysis was performed in GraphPad Prism v7 and statistical significance was considered for *p* < 0.05. Comparison between samples was performed by a nonparametric Mann-Whitney test or paired *t*-test and correlations were calculated with Spearman *r*-test.

#### Potential Applications

With this 3D model, employing individual patient-derived immune cells, several applications can be achieved, such as the study of the cross-talk between immune cells and breast cancer cells or a medium-throughput drug screening, in a precision medicine approach. Indeed, here we propose the use of this platform, precisely as a tool to test different immune-modulators to improve breast cancer treatments.

##### Drug Screening

To perform a drug screening, any soluble drug (e.g., chemotherapy compounds), immune checkpoint inhibitor (such as the PD-1/PD-L1 axis), agonist antibody or small molecule, etc. can be added to the 3D co-culture in order to either kill the cancer cells directly or to promote killing mediated by the immune cells. The optimal day to add the compound is on day 8 (see Figure 1), so that PBMCs are already infiltrated in the tumor-like structure. The effect of the molecule can be observed after 2 days (day 10 of culture). Alternatively, immune-modulators that can alter the behavior of the immune cells, can be added only to the PBMCs, before their addition to the 3D culture (day 6). In this case, other drugs (e.g., chemotherapeutic compounds) can be added in the same experiment on day 8, and the collective effect observed on day 10.

As a proof-of-concept, here we disclose the use of a canonical immune-modulator—PMA/ionomycin—that acts as a stimulator of the PBMCs, leading to their activation and potentiating their effector functions. This stimulus was added directly to the PBMCs at day 6. The effect of stimulated PBMCs on the viability of tumor cells was observed at day 10 by flow cytometry (see the section *techniques to characterize the spheroids*).

*Note: perform a dose-response curve to assess the optimal drug concentration for the screening*.

1. At day 6 of 3D co-culture:
  - Remove 100 μL of medium per well and discard.
  - *Note: remove the medium by placing the pipette tip with a 45*° *angle and aspirate from the top of the well, without touching the spheroid*.
  - Add 1000 PBMCs suspended in 100 μL of supplemented RPMI per well, previously stimulated with 35 ng/mL PMA and 1 μg/mL of ionomycin.
  - Incubate in a humidified environment at 37°C, 5% CO_2_.
2. At day 10:
  - Collect the spheroids by pipetting the total 200 μL from each well.
  - *Note: perform this step with a P1000 micropipette*.
  - Transfer the spheroid to a 1.5 mL microtube and disrupt the structure by pipetting up and down several times.
  - *Note: use at least 6 spheroids per condition*.
  - Centrifuge at 200 g for 5 min.
  - Remove the supernatant and resuspend the pellet in PBS 1X.
  - Perform the staining for Flow Cytometry.

### Anticipated Results

In this protocol, we reveal a reproducible, simple, quick, and economical method to construct 3D tumor-like structures with infiltrated immune cells derived from breast cancer patients (Figure 1). This method was developed for the cell line MDA-MB-231, normally described as challenging to form spontaneous 3D structures (19, 20).

This cell line is a representation of the most difficult to treat breast cancer subtype, which is triple negative breast cancer (TNBC). We have performed a characterization of several surface markers of MDA-MB-231 cells (Figure S1), following the Flow Cytometry methods in *Techniques* section. Interestingly, we observed that this cell line has a cancer stem cell-like phenotype (CD44^high^/CD24^low^), which can be correlated with less effective treatment response in breast cancer (24). This cell line is also positive for the immunosuppressive trait PD-L1, and CD47 (Figure S1), which can be seen as possible targets to develop directed therapies. PD-L1 is an immune checkpoint, which can inhibit the effector function of T cells; while CD47 might help the tumor cells to evade phagocytosis by phagocytic immune cells (25). The platform here developed, bearing embedded immune cells, could allow to test these and other several immune-modulator agents to develop new TNBC-specific therapies.

To validate our protocol, we have also performed a co-culture protocol with MCF-7 cell line, which is from the most frequent subtype of breast cancer (estrogen receptor), using a similar procedure. Although both cell lines share some features (Figure S1), such as the expression of CD47, MCF-7 lacks the expression of PD-L1 at its surface. The spheroids with this cell line were fully formed at day 3 (Figure S2).

MDA-MB-231 were seeded in round bottom plate in low-attachment conditions. A uniform spheroid of MDA-MB-231 is completely formed and compact at day 6 (Figure 2A); in addition, the spheroid formation was highly reproducible and none had their morphology compromised. Bright-field images were collected every 2 days (day 6, 8, and 10) and the spheroid area was analyzed with Image J. Briefly, an automatic threshold can be applied to the image, to reduce the operator bias (Image—Adjust—Threshold) and quantification of the area can be performed (Analyze—Measure). Interestingly, we observed that it increases from day to day (Figure 2B), showing the capacity of the cancer cells to divide and proliferate, which mirrors their wellbeing in the referred conditions and their capacity to maintain the 3D structure. Likewise, MCF-7 spheroids showed a similar growing pattern (Figure S2). Besides assessing spheroid area, other analysis can be performed by Image J, such as spheroid diameter, fluorescence quantification, circularity, to name a few.

After the 3D formation, we added breast cancer patient-derived PBMCs, in a 1:1 ratio, replicating more accurately than other reported systems, the *in vivo* likely proportion of immune cells to the tumor, as we have previously demonstrated (3). The characteristics of the patients enrolled in this study are described in Table S1. We observed that after 24 h only, the PBMCs were able to infiltrate the tumor-like structure (Figures 3A,B), without affecting the spheroid area (Figure 3C). This result was achieved by imaging techniques using a cell tracer to distinguish the immune cells (CellTracker™ Orange CMTMR) and another to distinguish tumor cells [carboxyfluorescein succinimidyl ester (CFSE)]). Additionally, if desired, the employment of the CFSE dye has the advantage of following the tumor cells' proliferation (the dye will become more diluted with every cellular division). Likewise, immunofluorescence for any kind of cell marker can be performed after spheroid fixation (see Imaging protocol in the Techniques section). Identical PBMCs' infiltration in the whole 3D conformation of the tumor-like structure was observed for MCF-7 spheroids (Figure S3).

Several techniques, besides imaging, could be applied to this 3D co-culture, such as immunophenotyping by flow cytometry, western blot, real-time qPCR, or ELISA of the culture supernatant, to name a few, to investigate in-depth how the immune cells of a particular patient react to cancer cells. For instance, it may help to understand which subpopulation of lymphocytes, among the effectors or regulatory ones, become more activated and able to proliferate; which cytokines are being produced or which receptors and co-receptors are being stimulated.

Besides showing great potential as a platform to study the complex interaction between cancer and the immune system of particular individuals, this system is particularly suited to access different strategies to immune-modulate in different patients the anticancer immune response.

Indeed, here we assessed the viability of the tumor cells, by flow cytometry, in the presence of patient-derived PBMCs with or without previous stimulation (Figure 4). Interestingly, we observed that the addition of PBMCs from the majority of breast cancer patients to the cancer cells spheroid reduces the viability of the tumor cells (*p* = 0.02, Figures 4A,B), showing the cytotoxic profile against cancer cells of patient-isolated PBMCs. However, PBMCs from certain patients barely affect the viability of cancer cells, suggesting that the range of the immune responses varies widely. This may be due to impaired activity of effector immune cells in patients where an immunosuppressive environment predominates. Therefore, we also used PBMCs previously stimulated *ex vivo* with the canonical stimulus of PMA/ionomycin and, after 4 days in co-culture, we observed that stimulated PBMCs reduced even further the viability of the MDA-MB-231 cells (*p* < 0.0001, Figures 4A,B), indicating that activated immune cells have a higher cytotoxic capacity. Furthermore, when comparing non-stimulated with the stimulated condition, there is a significant difference in their effect on tumor cells (*p* < 0.0001, Figure 4B). Notably, PBMCs from 16 breast cancer patients when PMA/ionomycin stimulated, similarly became more efficient in exerting their cytotoxicity towards cancer cells (Figure 4C). Interestingly, the same effect was observed for the co-culture with patient-derived PBMCs and MCF-7 tumor cell line (Figure S4), suggesting that immuno-modulating the PBMCs (at least with the canonical stimulus here employed) could improve their cytotoxicity against breast cancer lines with different characteristics.

The analysis of IFN-γ level in the co-culture, performed with the stimulated PBMCs, by ELISA, revealed a negative correlation between the viability of tumor cells in the co-culture and the quantity of released IFN-γ into the supernatant (Figure 4D and Figure S4D), in line with the idea that this cytokine is essential for the activity of cytotoxic T cells (26). Thus, the level of this molecule in the supernatant of the co-culture could also be a readout of the extension of the immune response toward the tumor cells.

Although PMA/ionomycin is a canonical stimulus, it is not compatible with clinical use. Nonetheless, the results here described suggests that we could indeed modulate the antitumor immune response and that we could use the 3D *in vitro* system here developed to identify which clinical grade stimulus is more indicated to boost the antitumor immune response of a particular patient. For instance, as the cell line here employed is PD-L1+, this system could be used to test the effect of anti-PD-1 or anti-PD-L1 alone or in combination with other drugs, such as immune-based small molecules or even chemotherapeutic agents, to address, in a precision medicine approach, the best treatment for each patient.

Here we demonstrate one of the applications of this protocol—to assess the effect of potential immune-modulators on individual PBMCs' capacity to eliminate tumor cells. Nevertheless, this protocol can be adapted for other scenarios, including different breast cancer cell lines or cell lines from different types of cancer. Additionally, although the allogeneic system here presented appears suitable to evaluate the antitumor response of patients' immune cells, we envisage that patient-derived tumor cells can also be incorporated in this protocol to obtain an eventually more clinical relevant autologous system. Nevertheless, modifications to the protocol have to be made to obtain a 3D patient-derived tumor culture—from a surgical specimen, a cell suspension has to be obtained (either by mechanical or enzymatic disaggregation) and cultured on 2D to expand. Expanded patient-derived tumor cells can then be added to agarose-coated wells and allow to form a 3D spheroid. The time from a 2D culture and the application of the primary tumor cells to a 3D conformation, as well as the time to obtain the 3D structure would have also to be optimized.

## Discussion

In this manuscript, we reveal an optimized method to form 3D spheroids with the breast cancer cell line MDA-MB-231 and patient-derived immune cells, specially foreseeing its application in the discovery of new immunotherapeutic strategies that could improve individually breast cancer patient care. The cell line here employed is particularly used to study the most difficult to treat type of breast cancer—triple negative breast cancer [TNBC, (21)]. Since TNBC has no specific molecular target, the main therapeutic strategy used for these patients is systemic chemotherapy, but the prognosis tends to be poorer when compared to other types of breast cancer.

An important role of the immune system is to identify and eliminate tumors. Tumor-associated antigens are recognized by immune cells that mount an immune response against tumor cells which may contribute to eliminate chemotherapy-resistant tumor cells. However, tumors develop several mechanisms to escape immune recognition. For instance, when T cells interact with tumors, they may deliver several potential inhibitory signals, including lack of proper co-stimulation and induction of immunosuppressive T regulatory cells (4). Therefore, an important challenge in cancer treatment is the identification of effective strategies for enhancing its clinical efficacy. The field of immuno-oncology has been rapidly growing and several immunological molecules have been exploited in the latest years to overcome the tumor's effort to evade the immune system, with great success in some types of cancers (e.g., melanoma, lung cancer). The immunotherapy could be achieved by blocking pathways that limit the immune response, such as the PD-1/PD-L1 axis, the CTLA-4 or the T regulatory cells; or using immune system stimulating agents, such as pro-inflammatory cytokines or other agonists of co-stimulatory receptors to enhance the activity of cytotoxic T cells (4).

Due to the unmet clinical need to find a target and an efficient therapy for TNBC, this type of cancer is the main breast cancer subtype enrolled in immuno-oncology studies (2, 21). Interestingly, here we observed that the cell line MDA-MB-231 (TNBC) besides exhibiting a cancer stem cell-like (CD44^high^/CD24^low^) phenotype, is also positive for PD-L1 and CD47 (Figure S1), two immune-related markers that can be further explored as new alternative target therapies. Actually, in cases of metastatic TNBC, immunotherapies against PD-L1 are already approved, namely Atezolizumab along with paclitaxel (27). Therapies against CD47 are also being applied in several clinical trials, although not specifically in breast cancer (25).

However, considering the wide range of antitumor immune responses, even against the same type of tumor, due to individual traits, it is important to further develop tailored treatments for TNBC patients. Regularly, only late-stage patients are given the opportunity to receive novel therapies, which might offer the potential for long-term disease control (although those therapies have shown benefits only in a small percentage of patients); while patients with less advanced disease never reached the chance of being treated with off label medications.

Thus, 3D cultures with patient-derived material provide a simple-to-use and rapid system to test *ex vivo* several new therapeutic strategies, that might be later translated into the patients. Although attempts have been made to successfully form spheroids with MDA-MB-231 cell line (19, 20), most use scaffold-based techniques. Here, we disclose a simple technique that allowed the formation of a uniform and compact 3D structure with this cell line, that has the advantage of being simpler, cheaper, and requiring only a few cells (1000) per well/condition. Indeed, Froehlich *et al* were able to form spheroids but only when 10,000 MDA-MB-231 cells were cultured per well in Matrigel (19) and, similarly, Ivascu *et al* achieved compact spheroids with this cell line when 5,000 or 10,000 cells were seeded per well. These last authors even screened several additives to increase 3D compaction (including collagen type IV, fibronectin, laminin, heparan sulfate proteoglycan, and chondroitin sulfate) but did not observe spheroid formation after 14 days. The 3D formation was only observed when reconstituted basement membrane was added to the culture. The use of agarose provides a non-adhesive structure with a concave surface shape and without cellular recognition sites, forcing the cells to aggregate and form compact 3D structures.

Furthermore, we included breast cancer patient-derived peripheral blood mononuclear cells (PBMCs) in the 3D culture to allow a representation of each individual immune response to the tumor and therefore obtain a platform appropriate to screen different strategies that could potentially engage the immune cells of a particular patient to recognize and eradicate tumor cells.

Indeed, we were able to build this co-culture in a 1:1 ratio, which is another advantage when compared to other 3D co-cultures with tumor and immune cells, where a hardly physiological, significantly higher [for instance 1:10 (23)], amount of immune cells is employed. Likewise, when using patient-derived material, the use of the minimum amount of cells is a gain. As we have previously observed, in breast cancer patients, the immune infiltration is never higher than 50% of the tumor mass (3), the 1:1 ratio here implemented was another step toward *in vivo* replication.

The method here described for the co-culture is straightforward. Once optimized the protocol, namely the time needed for the spheroid formation and the number of each cell population to use in the co-culture in order to reach the desired ratio of 1:1, we have encountered no significant limitations or challenges.

The functional readouts used were the viability of tumor cells (evaluated by flow cytometry after harvesting the spheroids and staining the cells with a viability dye and a pan-leucocyte antibody to distinguish immune from cancer cells) and the production of IFN-γ by ELISA. However, other evaluation methods could be employed. For instance, live imaging to visualize the immune infiltration or to assess tumor cell apoptosis; real-time qPCR to quantify the expression of genes related to cell death and to immune-activation cascades; western blot or immunofluorescence to analyze specific markers.

As a proof-of-concept of the application of the implemented 3D system, we used the canonical stimulus PMA/ionomycin to increase the cytotoxic profile of breast cancer patient-derived PBMCs. Indeed, we observed that stimulated PBMCs were able to reduce significantly the viability of MDA-MB-231 and produce more IFN-γ when previously stimulated, corroborating that these abilities could be modulated and that the established protocol creates a physiologically relevant environment for a medium-throughput screening of clinical grade immune-based compounds. For comparison, we used the estrogen receptor positive breast cancer cell line—MCF-7, and observed a similar effect of the patient-derived PBMCs on the tumor cells' viability.

**Figure 5:**
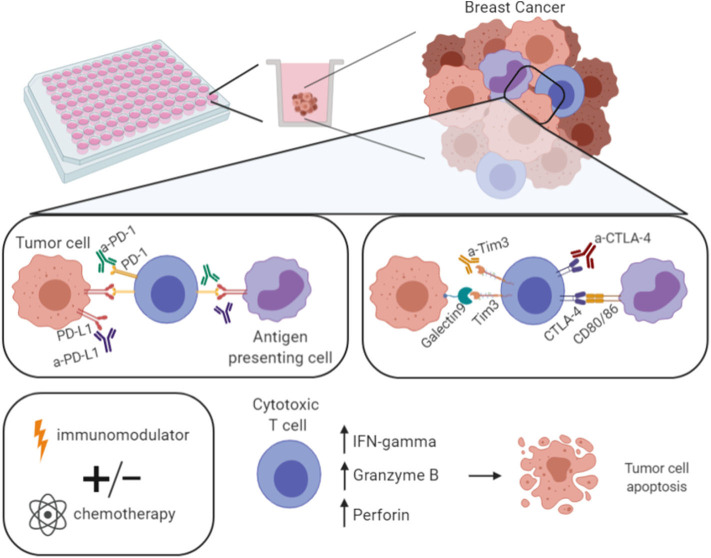
Model of the developed platform for drug screening. With the developed 3D co-culture system here described it is possible to perform a drug screening—multiple approaches per plate. For instance, the use of antibodies against checkpoint inhibitors (anti-PD-1, -PD-L1, -CTLA-4, -Tim3), or immune-modulators, such as PMA/ionomycin. These strategies can be used alone or in combination with standard chemotherapeutic agents with the ultimate goal of increasing the expression of IFN-γ, Granzyme B, and Perforin, hence the cytotoxic T cells effector function, in order to eliminate tumor cells. Figure performed in Biorender.

In fact, in the future, an aliquot of patients' blood could be immunophenotyped by flow cytometry to assess the expression of the inhibitory and/or stimulatory co-receptors present at the main T cell subpopulations, therefore checking the more probable targets. Then, based on this information, monoclonal antibodies designed to block inhibitory pathways such as the PD-1/PD-L1 axis or other immune checkpoints—CTLA4, Tim-3 (T-cell immunoglobulin domain and mucin domain 3) and LAG-3 (lymphocyte-activation gene 3)—or to stimulate stimulatory co-receptors—CD137, GITR (glucocorticoid-induced TNFR family related gene), OX40 and CD27—or small molecules, either agonist or antagonists, or cytokines could be incorporated in the 3D co-culture assay with the PBMCs from the same patient. Therefore, we can say that our platform is designed to develop immunotherapies in a “personalized medicine” way, even if the 3D cellular structure that mimics the tumor employs breast cancer cell lines, because the main target of the therapies to be tested are the individual patient-derived PBMCs, that are added in co-culture. Moreover, as it has been discussed in the field, the combination of immunotherapies with other drugs, namely chemotherapeutic agents, can also be evaluated on an individual level (Figure 5).

We anticipate that some strategies should induce the enhancement of the immune components that will result in the activation of the tumor-reactive cytotoxic T cells, and ultimately in the rise of IFN-γ and cytotoxic-related molecules, such as granzyme B and perforin, hence leading to more efficient tumor cell killing (Figure 5). Certainly, the best strategies would be different from patient to patient.

Due to the fact that the same quantity of PBMCs added to the tumor-like spheroid may include different percentages of cytotoxic T cells (depending on the patient), comparisons of the immune-mediated killing and the effect of compounds between different patients would be hard to establish, but we have to keep in mind that the system here developed aims essentially to screen potential immune-modulatory agents to increase the immune-mediate killing individually, and not to make comparisons between different individuals.

Moreover, the system proposed is an allogeneic system, meaning that the T cell receptor (TCR) of the patient-derived T cells do not match the major histocompatibility complex (MHC) molecules present at the tumor cell line. T cell's alloreactivity is now a well-established phenomenon and although its molecular basis remains enigmatic, it is becoming more clear that many T cells recognize alloantigens with high specificity (28). Thus, interactions of tumor spheroids with T cells and the impact of immune-modulators on tumor spheroids fate are being explored through allogeneic co-cultures (23), as the establishment of autologous platforms would represent additional challenges. Indeed, our system also demonstrated that the immune cells harvested from patients can recognize and destroy tumor cells independently of TCR/MHC matching, and that this ability could be improved by proper stimulation. The alloreactivity, at least of some T cells may explain why this is a suitable system to allow a screening of immune based compounds with potential to improve T cells cytotoxicity.

Although optimizations must be performed for each patient/agent (or combination of agents) tested, namely in terms of the concentration range, which can be time-consuming, this protocol is economic and easy to handle, allowing the screening of several strategies in one single plate and it can be implemented in any laboratory with cell culture facilities. Though here we used patient-derived PBMCs regardless of the breast cancer subtype of the donors, in the future this established model with MDA-MB-231 cell line should be used to test immune-modulators for PBMCs derived from a patient with TNBC. In addition, this protocol is perfectly suited to be used with PBMCs from other sources and can be adjusted for different culture times and drug concentration to adapt to each assay need.

Expectedly, the use of this 3D co-culture in pre-clinical tests will contribute to the implementation of novel and more tailored therapies that could ameliorate individual breast cancer care.

## Data Availability Statement

All datasets generated for this study are included in the article/Supplementary Material.

## Ethics Statement

The studies involving human participants were reviewed and approved by Ethical committee of Hospital CUF Descobertas, Ethical committee of Hospital de Vila Franca de Xira, and Ethical committee of NOVA Medical School, Faculdade de Ciências Médicas da Universidade Nova de Lisboa. The patients provided their written informed consent to participate in this study.

## Author Contributions

DS conducted all the experiments, analyzed and interpreted the data, performed the statistical analysis, assembled all the figures, and wrote the manuscript. AM performed the flow cytometry analysis to characterize MDA-MB-231 and MCF-7 cell lines, in terms of surface markers, and revised the manuscript. SB contributed to scientific discussion, helped in the obtainment of patients' samples, and clinical data. AJ contributed to scientific discussion and revised the manuscript. MC designed the experiments, supervised the study, interpreted the data, and wrote the manuscript. All authors approved the final manuscript.

## Conflict of Interest

The authors declare that the research was conducted in the absence of any commercial or financial relationships that could be construed as a potential conflict of interest.
