# Supplementary material for: Establishment of a 3D Co-culture With MDA-MB-231 Breast Cancer Cell Line and Patient-Derived Immune Cells for Application in the Development of Immunotherapies
Source: Front Oncol. 2020 Aug 27;10:1543. doi: 10.3389/fonc.2020.01543 (PMC7482668; doi:10.3389/fonc.2020.01543)
Supplement: Supplementary file 1 [file Data_Sheet_1.docx]

Supplementary Material


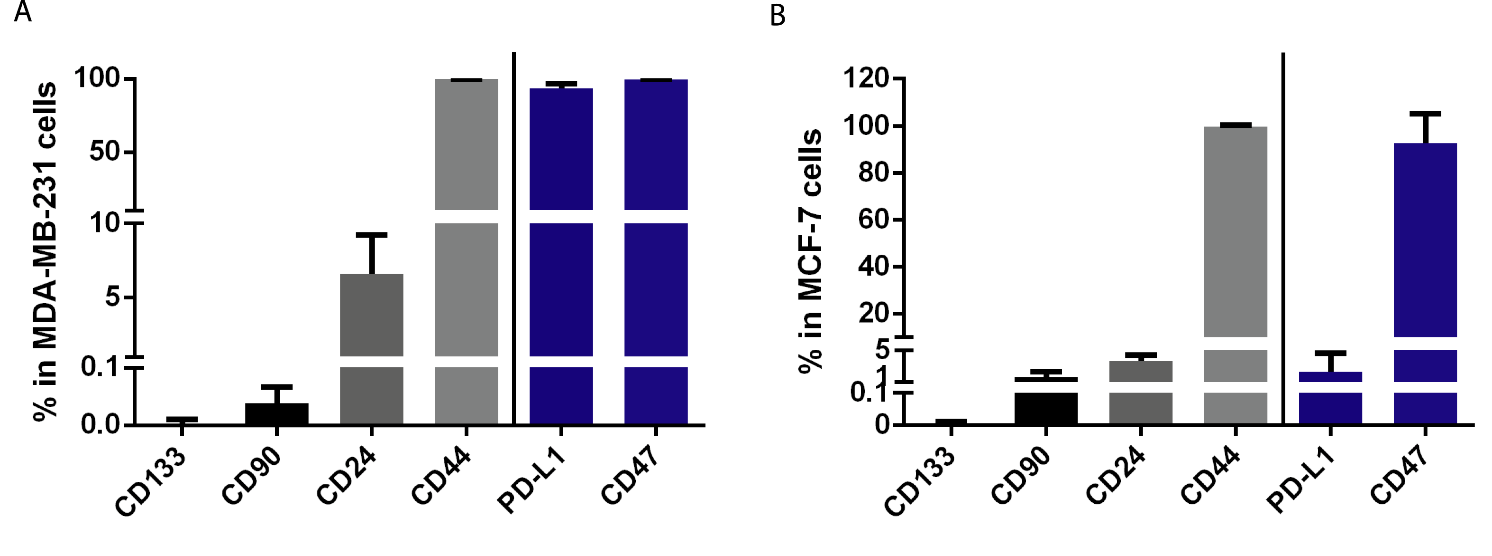


Figure S1 – **MDA-MB-231 cells have a CD44high/CD24low phenotype and are positive for PD-L1 and CD47**. **(A)** Characterization, by flow cytometry, of the stemness markers CD133, CD90, CD24 and CD44 (in gray); and of the immune markers PD-L1 and CD47 (in blue) in MDA-MB-231 cells. **(B)** For comparison, the same characterization by flow cytometry was performed in MCF-7 cells. Data is represented as mean ± standard deviation (n=3).


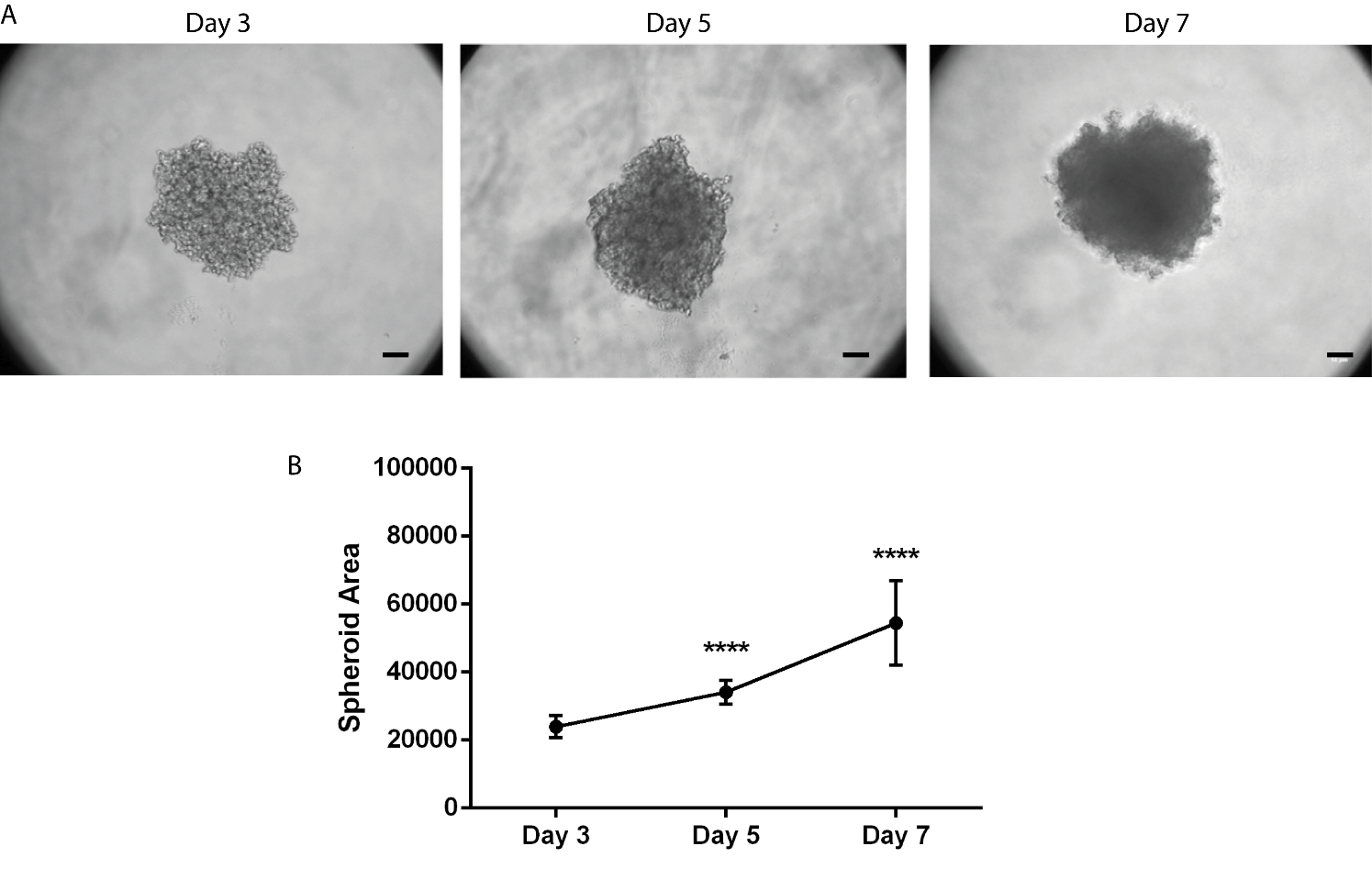


Figure S2 - **Area of the** **3D spheroids with MCF-7 breast cancer cell line** **increases with time.** **(A)** Bright-field images of MCF-7 spheroids at days 3, 5 and 7 (spheroid was completely formed at day 3). Scale bars – 50 μm. **(B)** Spheroid area of MCF-7 spheroids at day 3 (n=9), day 5 (n=8) and day 7 (n=9). Data is represented as mean ± SD, ****p<0.0001 (paired t-test).

Table S1 – Patient characteristics (age and body mass index) and the subtype of breast cancer.

| Age | Median: 57 (range: 33-66) | |
| --- | --- | --- |
| Body Mass Index | Normal weight (BMI 18.5-24.9) | 45.45% |
|  | Overweight (BMI 25-29.9) | 27.28% |
|  | Obese (BMI >30) | 27.27% |
| Subtype of Breast Cancer | Estrogen receptor (ER+) | 45.46% |
|  | HER2+ | 18.18% |
|  | Triple Negative Breast Cancer (TNBC) | 36.36% |


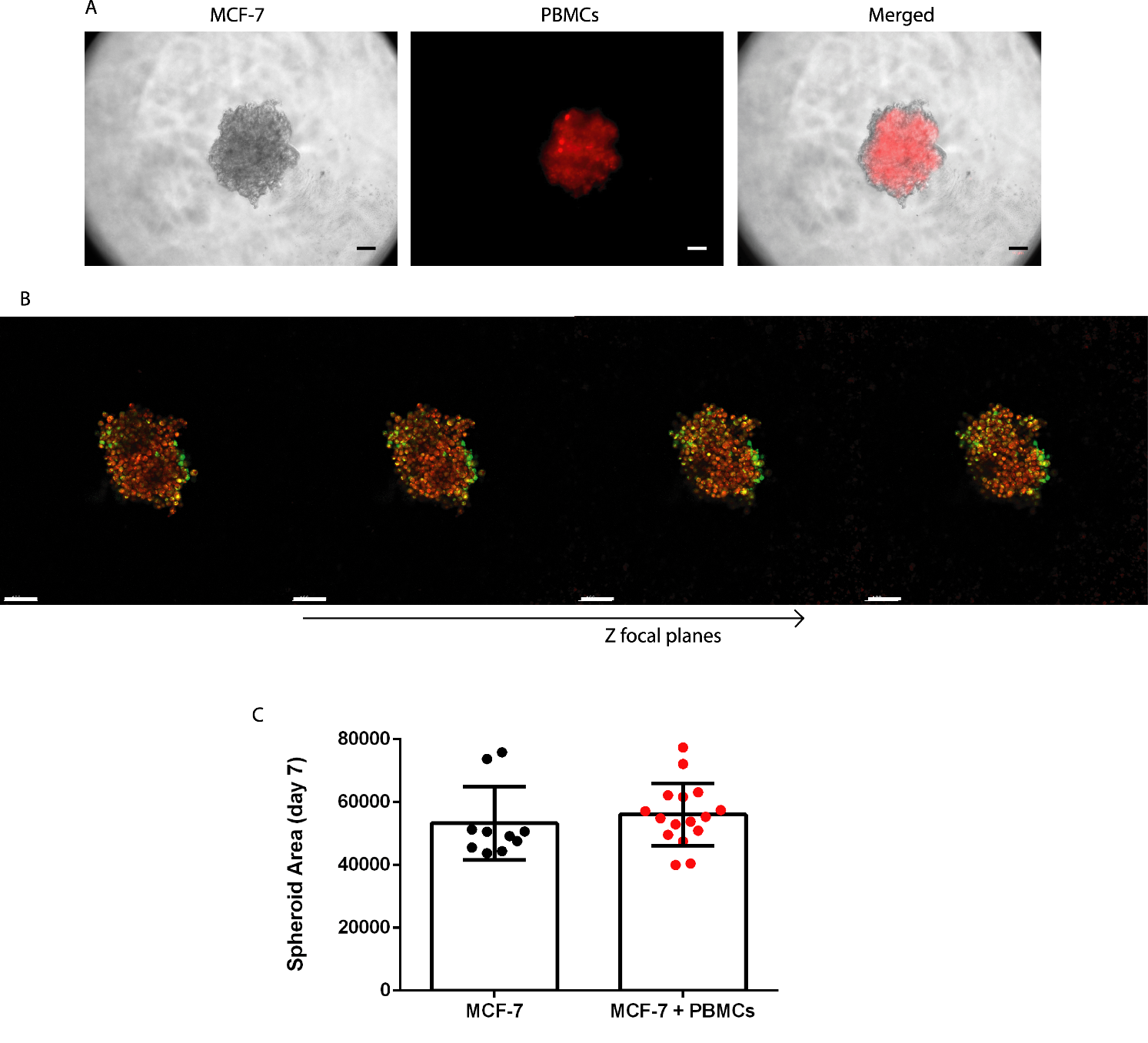


Figure S3 **- Patient-derived immune cells are able to infiltrate the 3D spheroid of MCF-7 breast cancer cell line. (A)** Bright field of MCF-7 spheroid (left panel), patient-derived PBMCs stained in red with a cell tracer dye (middle panel) and the two photos merged (right panel), at day 4 of the culture (24h after the addition of the PBMCs); 10x objective, scale bar 50 μm. **(B)** Confocal images of 3D spheroids of MCF-7 cell line (stained in green with CFSE) and patient-derived PBMCs (stained in red with a cell tracer dye), at day 4 of culture (24h after the addition of the PBMCs). Stills were acquired with 10x objective for different Z focal planes, to demonstrate the immune infiltration in a 3D structure; scale bar 100 μm. **(C)** Spheroid area of MCF-7 cells in monoculture (n=10) and in co-culture with breast cancer patient-derived PBMCs (MCF-7 + PBMCs, n=16) at day 7 of the culture (4 days after the start of the co-culture). Data are represented as mean ± SD.


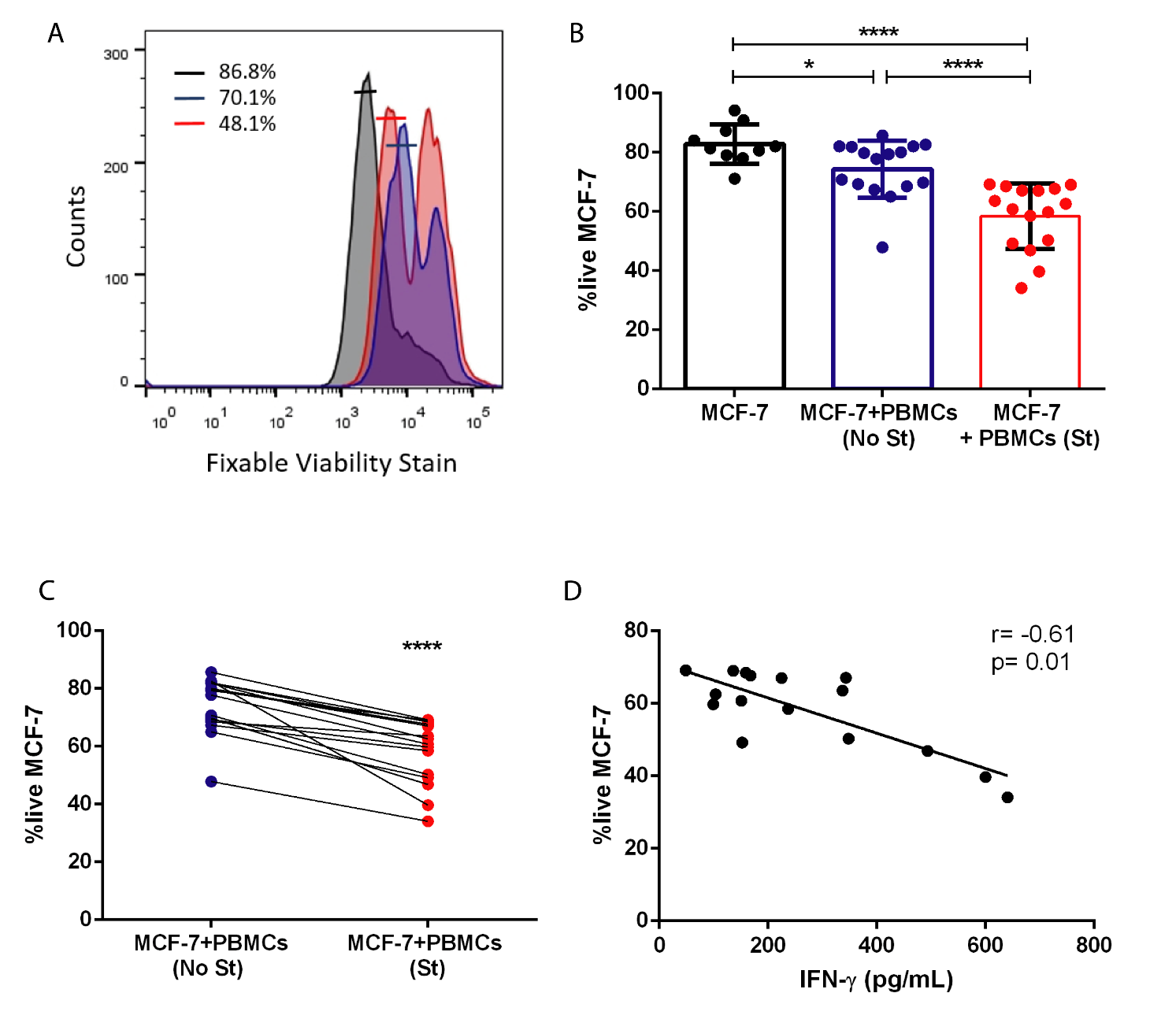


Figure S4 – **Previous stimulation increases the cytotoxicity of patient-derived immune cells against MCF-7 tumor cells**. **(A)** Representative histogram of the flow cytometry analysis of the viability of MCF-7 in monoculture (black line), in co-culture with non-stimulated PBMCs (blue line) and in co-culture with previously stimulated PBMCs (red line); the percentage of live cells in each condition is presented. **(B)** Percentage of viable breast cancer cells (MCF-7) in monoculture (black bar, n=10), in co-culture with non-stimulated PBMCs (+PBMCs (No St), blue bar, n=16) and in co-culture with PMA/ionomycin stimulated PBMCs (+PBMCs (St), red bar, n=16). **(C)** Data on the viable tumor cells in co-culture (same as in B) represented as connected dots, to highlight the effect of PBMCs stimulation in individual cases. **(D)** Correlation (Spearman r) between the viability of the MCF-7 cell line and the production of IFN-γ, detected in the co-culture supernatant and quantified by ELISA. Data was collected at day 7 of the culture (4 days after the start of the co-culture) and it is represented as mean ± SD. Mann-Whitney test was applied in (B) and a paired t-test was used in (C), *p<0.05, ****p<0.0001. All the wells have the same quantity of DMSO, as this reagent was used to prepare ionomycin.
